# 

**Published:** 1856-07

**Authors:** 


### RECEIPTS ON SUBSCRIPTIONS,

FROM MAY 20TH TO JUNE 23D, 1856.

### ILLINOIS.

| | | | |
|---|---|---|---|
| Dr. J. B. Meigs, | Vol. 5, | $2 00 |
| " R. T. Goodwin, | " 5, | 2 00 |
| " P. S. Secor, | " 5, | 2 00 |
| " C. M. Robertson, | " 5, | 2 00 |
| " E. R. Boardman, | " 5, | 2 00 |
| " J. H. Lawrence, | " 5, | 2 00 |
| " W. A. Hillis, | " 4, | 2 50 |
| " J. Barber, | " 5, | 2 00 |
| " F. B. Haller, | " 5, | 2 00 |
| " J. G. Tilden, | " 5, | 2 00 |
| " A. H. Luce, | " 5, | 2 00 |
| " J. R. Abell, | " 5, | 2 00 |
| " McMillan Marshall, | " 5, | 2 00 |
| " Geo. Higgins, | " 4, | 2 00 |
| " G. V. Ewing, | " 5, | 2 00 |
| " M. B. Van Patton, | " 5, | 2 00 |
| " J. D. Mitchell, | " 5, | 2 00 |

### INDIANA.

| | | |
|---|---|---|
| Dr. Jerry Wooden, | Vol. 5, | $2 00 |
| " W. F. Boone, | " 5, | 2 00 |
| " B. F. West, | " 5, | 1 00 |
| " C. H. Weaver, | " 5, | 2 00 |

### IOWA.

| | | |
|---|---|---|
| Dr. W. G. Elder, | Vol. 4, | $2 00 |

### WISCONSIN.

| | | |
|---|---|---|
| Dr. Ira Manley, Jr., | Vol. 2, 3 & 4, | $7 00 |
| " A. Bodenstab, | Vol. 5, | 2 00 |

### MICHIGAN.

| | | |
|---|---|---|
| Dr. G. B. Davidson, | Vol. 1, | $1 00 |

